# Hemodynamic Responses Evoked by Neuronal Stimulation via Channelrhodopsin-2 Can Be Independent of Intracortical Glutamatergic Synaptic Transmission

**DOI:** 10.1371/journal.pone.0029859

**Published:** 2012-01-10

**Authors:** Nadia A. Scott, Timothy H. Murphy

**Affiliations:** 1 Kinsmen Laboratory of Neurological Research, Department of Psychiatry, University of British Columbia, Vancouver, British Columbia, Canada; 2 Brain Research Centre, University of British Columbia, Vancouver, British Columbia, Canada; 3 Department of Cellular and Physiological Sciences, University of British Columbia, Vancouver, British Columbia, Canada; The Research Center of Neurobiology-Neurophysiology of Marseille, France

## Abstract

Maintenance of neuronal function depends on the delivery of oxygen and glucose through changes in blood flow that are linked to the level of ongoing neuronal and glial activity, yet the underlying mechanisms remain unclear. Using transgenic mice expressing the light-activated cation channel channelrhodopsin-2 in deep layer pyramidal neurons, we report that changes in intrinsic optical signals and blood flow can be evoked by activation of a subset of channelrhodopsin-2-expressing neurons in the sensorimotor cortex. We have combined imaging and pharmacology to examine the importance of glutamatergic synaptic transmission in this form of neurovascular coupling. Blockade of ionotropic glutamate receptors with the antagonists CNQX and MK801 significantly reduced forepaw-evoked hemodynamic responses, yet resulted in no significant reduction of channelrhodopsin-evoked hemodynamic responses, suggesting that stimulus-dependent coupling of neuronal activity to blood flow can be independent of local excitatory synaptic transmission. Together, these results indicate that channelrhodopsin-2 activation of sensorimotor excitatory neurons produces changes in intrinsic optical signals and blood flow that can occur under conditions where synaptic activation of neurons or other cells through ionotropic glutamate receptors would be blocked.

## Introduction

The link between neural activity and cerebral blood flow – neurovascular coupling – appears from the outset to be clearly delineated: initial energy demand in neurons drives localized oxygen consumption and the subsequent influx of oxygenated blood [1], [2]. While functional imaging techniques are predicated on this facile assumption, caveats must be placed when using changes in blood oxygenation and flow as proxies of neuronal activation, as it is not always clear which cell types – excitatory neurons, inhibitory interneurons, or glial cells – can initiate neurovascular coupling. It remains further unclear which pathways underlie the coupling of activity to changes in blood flow, with strong proponents both for neuronal and astrocytic intermediaries [1], [2].

Thus far, studies of neurovascular coupling have been limited by the inability to selectively initiate neuronal activity. In this study, we have investigated whether direct *in vivo* stimulation of excitatory neocortical neurons is sufficient to produce measurable changes in hemodynamic signals and blood flow. To achieve this, we have used channelrhodopsin-2 (ChR2) transgenic mice (B6.Cg-Tg(Thy1-COP4/EYFP)18Gfng/J) that express a photoactivatable non-specific cationic channel [3], [4], allowing us to selectively activate regions of the sensorimotor cortex [5]. These mice express ChR2 in deep layer cortical pyramidal neurons under the Thy-1 promoter with a pattern that closely resembles other animal lines with expression in predominantly cortical layer 5B neurons [5], [6]. While it has been recently demonstrated that activation of ChR2 expressing excitatory neurons evokes changes in the fMRI BOLD signal [7], [8], the underlying physiology and direct relationship of these responses to blood flow has not been investigated in detail. Furthermore, previous work has shown that intracortical microstimulation using electrodes can be used to examine regional connectivity by monitoring hemodynamic responses with fMRI [9], [10]. To this end, we have coupled pharmacology with selective neuronal activation to examine the role of glutamatergic signaling in the production of hemodynamic responses. Specifically, we have addressed whether activation of deep layer neurons alone is sufficient to initiate hemodynamic responses. Furthermore, using pharmacology we have examined the roles of ionotropic glutamate receptors and Group I metabotropic glutamate receptors in the production of ChR2-evoked hemodynamic responses. Our results indicate that hemodynamic responses can be initiated by activity within deep layer pyramidal neurons without the involvement of synaptically activated neurons or glia through ionotropic glutamate receptors.

## Methods

### Ethics statement

All animal procedures were performed in accordance with and approved by the University of British Columbia Animal Care Committee protocols (protocol #A09-0665) in accordance with the Canadian Council for Animal Care guidelines.

### Animals and surgery

Adult ChR2 transgenic mice (line 18, stock 007612, strain B6.Cg-Tg (Thy1-COP4/EYFP)18Gfng/J; the Jackson Laboratory, Bar Harbor, ME) were initially anaesthetized with isoflurane (4% in air for induction, 1.5% for surgery) and craniectomized over the right sensorimotor cortex as previously described [11]. In experiments involving no cortical application of pharmacological agents, the exposed brain was covered with 1–1.5% agarose (Type 3-A Sigma; A9793) dissolved in a HEPES-buffered (pH 7.3) physiological salt solution (135 mM NaCl, 5.4 mM KCL, 1 mM MgCl_2_, 1.8 mM CaCl_2_, and 5 mM HEPES) and sealed with a glass coverslip. Following surgery, isoflurane was discontinued and anaesthesia was maintained with ketamine (20 mg/ml)/xylazine (1 mg/ml), administered as necessary to maintain a constant level of anaesthesia; animals were supplemented with a mixture of oxygen in air. Temperature (37±0.5°C) was maintained throughout the experiment using a feedback-regulated heating pad monitored by a rectal thermometer. Heart rate and blood oxygen saturation were monitored (Starr Life Sciences pulse oximeter, Oakmont, PA).

### Optical imaging and stimulation

For photostimulation of ChR2-expressing neurons, we used a 473 nm photoactivation laser (CrystaLaser BCL-473-050, Reno, NV) as previously described [5]. For forepaw electrical stimulation, a train of pulses was delivered by a stimulus isolator (World Precision Instruments, Sarasota, FL) through two 30-gauge needles inserted subcutaneously into the left forepaw. Our stimulus protocol (1.5 mA, 3 Hz, 1–4 s) was adopted from the literature [12].

**Figure 1:**
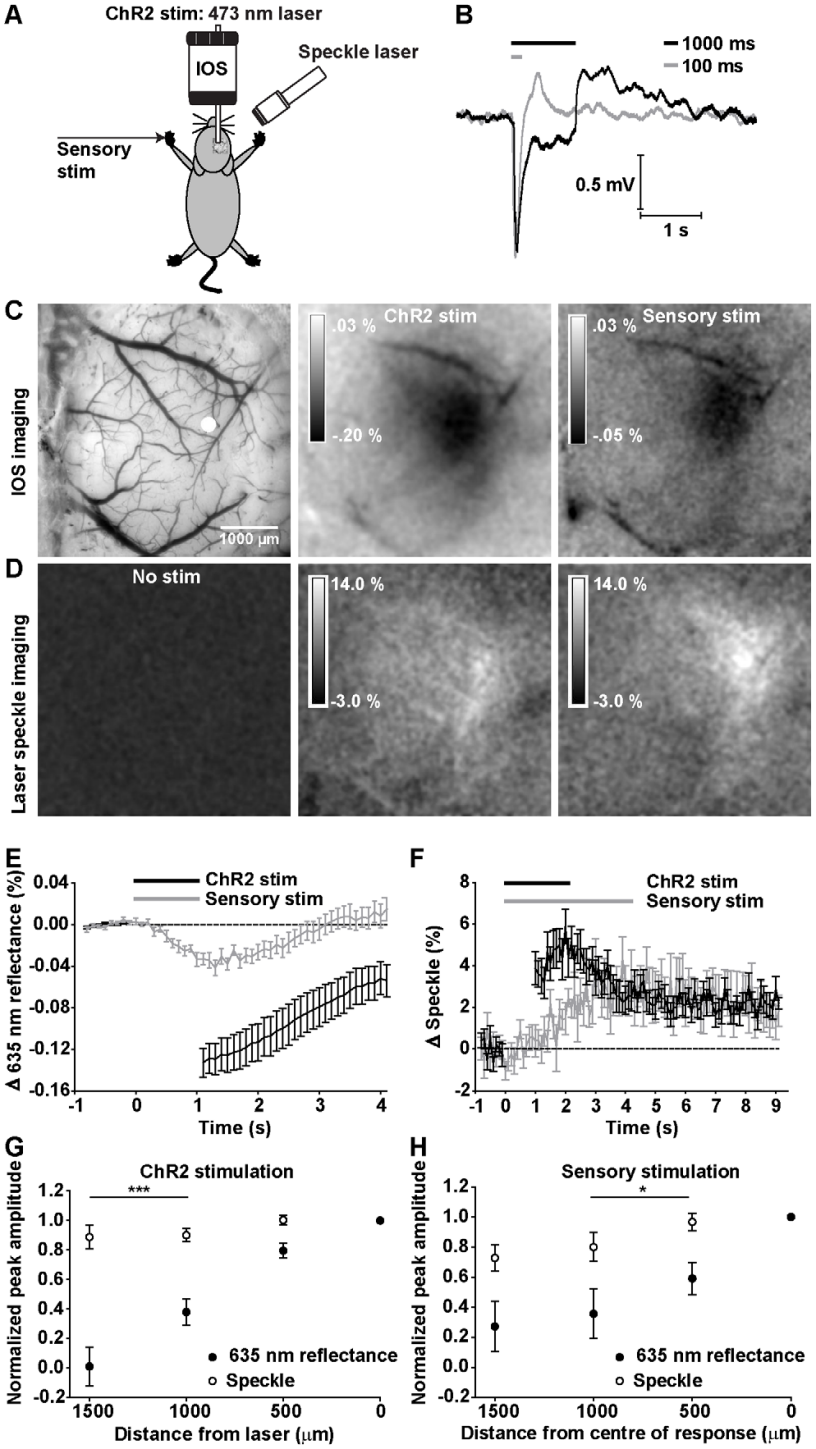
IOS and laser speckle imaging reveals ChR2 and sensory (forepaw) evoked hemodynamic responses. A, Schematic of experimental set-up. Responses to ChR2 stimulation (473 laser activation) and electrical sensory stimulation were recorded via IOS imaging (635 nm) and laser speckle imaging (785 nm). B, Averaged EEG responses (20 trials) to 100 ms and 1000 ms of ChR2 stimulation in a representative animal. C, *left*: Green light image of the cortical surface. Peak IOS response (averaged over 1 s from peak) to 1 s of ChR2 stimulation (*middle*) and 1 s of sensory stimulation (*right*). D, Maximum normalized laser speckle contrast response (averaged over 1 s at peak) to no stimulation (*left*), 1 s of ChR2 stimulation (*middle*) and 4 s of sensory stimulation (*right*) in the same animal. C,D Data shown are averaged 20 trials from a representative animal. E, Temporal profiles of IOS responses evoked by 1 s of ChR2 stimulation (n = 23) compared to 1 s of sensory stimulation (n = 12). F, Temporal profiles of laser speckle responses evoked by ChR2 stimulus train durations of 1 s (n = 13) compared to 4 s of sensory stimulation (n = 12). B,E–F Grey and black bars represent onset and duration of stimulation. Blanked data in temporal profiles reflect laser stimulus artifact. The peak amplitudes of ChR2-evoked IOS and speckle contrast responses were compared as a function of distance from the centre of laser activation (G). The spatial extents differed within animals at distances greater than 1000 µm (n = 6, both p<0.001, paired t-test). H, A similar effect was observed in sensory-evoked IOS and speckle contrast responses at distances of 500 and 1000 µm (n = 4, both p<0.05, paired t-test). Error bars represent SEM.

To visualize the cortex and vasculature, the surface of the brain was illuminated with green LEDs, while red LEDs (centred at 635 nm) were used for intrinsic optical imaging (IOS), with the depth of focus set to approximately 250 µm to blur the contribution of surface blood vessels (set-up shown in Figure 1A). Intrinsic optical signal imaging allows the measurement of oximetric signals through the back-reflection of light [13]; at the wavelength used in the present study, the measurements comprise both changes in the oxygenation state of hemoglobin and blood volume [14]. All images were captured with a CCD Dalsa 1M60 camera, with image acquisition performed using EPIX XCAP software (Buffalo Grove, IL; v2.2) with an E1DB frame grabber (EPIX). For IOS, each run consisted of twenty trials separated by a 45 s intertrial interval to allow responses to return to baseline. For each trial, 50 frames over 5 s were obtained at 10 Hz. These images were summed over repeated trials, from which the percentage change in 635 nm reflectance (ΔI/I %) was calculated at 32-bit resolution by dividing the difference between pre- and post-stimulation images by pre-stimulation images using a custom-written ImageJ (NIH, Bethesda, MD) plugin [15]. Due to the oversaturation caused by the 473 nm stimulation laser, these frames were blanked; thus, blanks in the displayed temporal profiles of IOS responses reflect stimulus artifacts. The terms ‘changes in 635 nm reflectance’ and IOS responses are used interchangeably throughout the text.

To assess blood flow, we used laser speckle contrast imaging, which capitalizes on the flow of blood cells that causes blurring of interference patterns created by coherent light illumination [16], [17]. Illumination was provided by a 784 nm 32 mW StockerYale SNF-XXX_885S-35 laser (Stocker & Yale, Salem, NH) with an Edmund anamorphic beam expander T47274 (Edmund Optics, Barrington, NJ) held on a micromanipulator at a 30° angle (Figure 1A). A polarizer filter was used to modulate laser output. As with the IOS data, laser speckle images were saturated during periods of 473 nm laser stimulation and were blanked accordingly. However, in later experiments, a 715 nm lowpass filter was used to prevent light from the 473 nm laser from entering the camera. Data with and without the lowpass filter were included in the present study; thus, some figures do not have a stimulus artifact. For stimulation trials, each run consisted of twenty trials separated by a 45 s intertrial period, with 100 images collected over 10 s (10 Hz) using 10 ms exposure. Spatial laser speckle contrast was calculated using ImageJ (NIH) as described previously [18] [11]. Here, we additionally took the inverse square of the laser speckle contrast as an approximation of blood flow [19]. Thus, it is important to note that with this calculation, an increase in speckle contrast (lighter values) reflects increased blood flow. The terms changes in blood flow and ‘speckle contrast’ are used interchangeably throughout the text.

### Imaging analysis

For IOS and laser speckle data, temporal profiles were derived from ROIs (100×100 pixels) centered on the site of laser activation; profiles were smoothed (adjacent averaging) over 1 s prior to identifying the peak (defined as the first peak following the end of stimulation). Images for figures were Gaussian filtered (radius in pixels = 3 for IOS, 8 for speckle contrast) and speckle contrast images were averaged over 10 frames in ImageJ.

### EEG

Electrophysiological data were obtained in parallel with imaging sessions to compare stimulation-evoked EEG responses to hemodynamic changes. We placed a Teflon-coated silver wire on the surface of the cortex, with the reference electrode placed subcutaneously in the nose. The signal was amplified and filtered (0.1–1000 Hz) using a differential AC amplifier (Model 1700, A-M Systems, Sequim, WA), with data collected using Clampex 9.2 (Molecular Devices, Inc., Sunnyvale, CA). Records were normalized and averaged using Clampfit 9.2 (Molecular Devices, Inc., Sunnyvale, CA). For spontaneous data, power spectrum analysis was performed using Clampfit 9.2. Frequency band analysis was performed to calculate the percentage of total power for the following defined frequency bands (Delta = 0.3–3 Hz; theta = 3–5 Hz; alpha = 8–15 Hz; beta = 15–30 Hz; and gamma 30–80 Hz).

### Pharmacology

To block local NMDA and AMPA receptors, we directly co-applied the ionotropic glutamate receptor antagonists CNQX (Sigma-Aldrich Co., Oakville, ON) and MK801 (Sigma-Aldrich Co.) to the surface of the intact dura (no agarose was present), at concentrations that we have previously demonstrated as sufficient to block sensory stimulation-evoked IOS maps (4.5 mM and 0.3 mM, respectively [20], both in physiological saline solution); responses were found to be similar whether or not the dura was present. For our experimental procedures, animals were pre-imaged, incubated for a minimum time of 30 min with CNQX/MK801 and post-imaged. Antagonists were reapplied to the cortex following each imaging run. To inhibit the Group I metabotropic receptor mGluR5, animals were pre-imaged, injected i.p. with MPEP (30 mg/kg; Tocris Bioscience, Ellisville, MO) and post-imaged after 20 min (for ChR2 stimulation experiments) and 40 min (for electrical forepaw stimulation experiments) in the same animals.

### Statistics

All statistics were performed in GraphPad Prism (GraphPad Software, Inc.). All *t*-tests performed are two-tailed. All ANOVA are two-way repeated measures, unless otherwise noted. Data are reported as mean ± SEM; n = number of animals. Statistical significance on all figures uses the following convention: * p>0.05, ** p>0.01, *** p>0.001.

## Results

To assess whether activation of subsets of ChR2-expressing neurons were sufficient to produce observable hemodynamic responses, we employed intrinsic optical signal (IOS) imaging [14], [21] and laser speckle imaging [16], [17] to observe signals that reflect changes in oxygenation and blood flow *in vivo*, respectively. Local targeting of blue collimated laser light (473 nm) through a coverslip-enclosed craniotomy allowed us to selectively stimulate ChR2-expressing neurons (Figure 1A) in the sensorimotor cortex with a high degree of spatial precision [5]. To compare these results with sensory stimulation, mice were given a brief shock to their forepaw. Successful activation by ChR2 stimulation was confirmed by a light-evoked EEG response (Figure 1B), while parallel local changes in (apparent) oxygenation were assessed by measuring changes in the reflectance of 635 nm light, a wavelength that is sensitive to intrinsic optical signals (IOS) of oxyhemoglobin and deoxyhemoglobin (Figure 1C). We observed relative changes in IOS upon presentation of 1000 ms (n = 23 mice) trains of blue light stimulation (100 Hz stimulation, 5 ms pulse duration) that paralleled responses evoked by forepaw (sensory) stimulation (1000 ms, 3 Hz, 300 µs pulse duration) in their time course (n = 12 mice) (Figure 1E). IOS responses could be reliably evoked with as little as a single 5 ms blue light pulse (not shown). Both ChR2-evoked changes in EEG and IOS could also be evoked through a thinned skull preparation (data not shown) at amplitudes comparable to those evoked in craniectomized animals [22]. To investigate whether functional hyperemia (elevated blood flow) were associated with these apparent changes in oxygenation (IOS signals), we used laser speckle contrast imaging at 785 nm to monitor changes in blood flow over large areas (Figure 1D). We observed changes in normalized speckle contrast upon presentation of 1000 ms (n = 13) trains of ChR2 stimulation, indicating that ChR2 stimulation could evoke changes in blood flow that were of equal or greater magnitude to those evoked by 4 s of forepaw stimulation (as 1 s of forepaw stimulation did not produce consistent responses, 4 s were used) (n = 12) (Figure 1F). Changes in speckle contrast could be reliably evoked with a 10 ms train of ChR2 stimulation (not shown). For both IOS and speckle contrast responses, temporal profiles were blanked during periods of laser stimulation.

To examine whether ChR2-evoked IOS and blood flow responses differ in their spatial extent when measured in the same animals, we compared the peak amplitude of responses at increasing distances (500 µm increments) from the centre of activation (0 µm), which we defined as the area of ChR2 laser stimulation (Figure 1G). Laser speckle and IOS time courses were smoothed (adjacent averaging) over 1 s prior to identifying the peak (see Methods). The peak amplitudes at each distance were normalized within each animal to peak amplitude at the site of stimulation prior to averaging over animals (n = 6). For ChR2-evoked hemodynamic responses, our data indicate that IOS responses were localized to the site of laser activation, while changes in speckle contrast were significantly more broader (Figure 1G; F(1,123) = 43.71, p<0.0001, 2-way ANOVA, n = 6). There was a significant difference between the spatial extents of IOS and speckle contrast responses at distances greater than 1000 µm (all p<0.001, post-hoc Bonferroni). A similar effect was observed when we compared the spatial extent of sensory (forepaw) stimulation-evoked IOS and speckle contrast responses measured in the same animals (Figure 1H; F(1,64) = 11.59, p = 0.0012, 2-way ANOVA, n = 4), with significant differences in the spatial extent of IOS and speckle contrast responses observed at distances of 500 µm and 1000 µm (both p<0.05).

ChR2 stimulation in ChR2-negative mice produced no observable change in EEG, IOS or speckle contrast signals, indicating that these effects are specific to mice expressing channelrhodopsin (Figure S1A–C). For these control experiments, negative littermates of ChR2-positive animals were used (n = 2). Responses in negative animals were similar to those observed in dead ChR2-positive animals (Figure S1D, S1E, n = 2).

In a small number of animals, stimulation experiments were performed under different anaesthesic conditions in order to determine whether the observed hemodynamic responses were specific to ketamine anaesthesia. While IOS responses could be evoked from ChR2 stimulation under 1.5% isoflurane anaesthesia in preliminary work, the evoked changes in speckle contrast were smaller than those obtained under ketamine xylazine anaesthesia (n = 2). Thus for the present study, ketamine xylazine anaesthesia was preferred over isoflurane as greater changes in IOS and speckle contrast could be more reliably elicited and this regime permitted comparisons to previous motor mapping work with ChR2 [5].

To test for possible negative effects of ChR2 laser stimulation on the cortex, we focused the laser on the forepaw sensory cortex map and examined the amplitudes of sensory-evoked IOS maps before and after 20 trials of light stimulation of ChR2. No differences in temporal profiles (Figure S2A; F(1,200) = 2.37, p = 0.1253) and peak amplitudes of evoked sensory maps before (ΔI/I −0.026±.004%) and after (ΔI/I −0.031±0.004%) ChR2 stimulation were found (Figure S2B; n = 5, p = 0.3463, paired t-test). In order to determine the effect of ChR2 stimulation on resting neural activity, we examined the power spectra of spontaneous EEG activity recorded (10 min) immediately prior to and following 20 trials of ChR2 stimulation (100 Hz, 1 s duration). As shown in Figure S2C, ChR2 stimulation had no significant effect (F(1,1330) = 0.04, p = 0.8400, n = 6) overall, nor when the data were compared by frequency band (Figure S2D; Bonferroni post-test, all bands p>0.05). Likewise, ChR2 stimulation had no significant effect on either heart rate or oxygen saturation measurements (Bonferroni post-test, both p>0.05) during 20 trials of ChR2 stimulation in comparison to measurements obtained prior to ChR2 stimulation (Figure S2E; repeated measures ANOVA, F(1,6) = 0.01, p = 0.9384).

**Figure 2:**
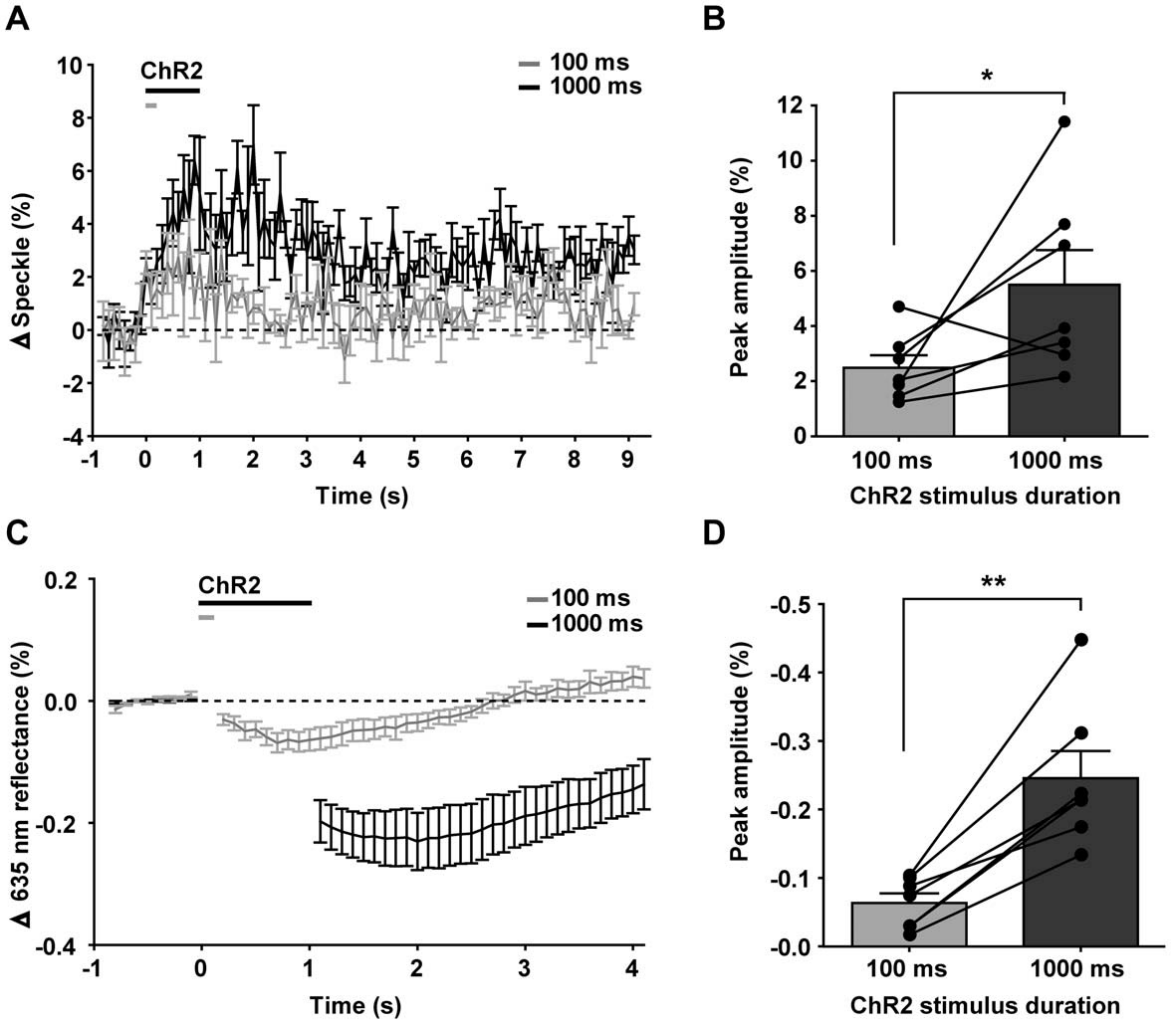
Effect of varying ChR2 stimulus train duration on evoked hemodynamic responses. A, Temporal profiles of laser speckle responses evoked by 100 ms (grey, n = 7) and 1000 ms of ChR2 stimulation (black, n = 7). B, Peak amplitudes of temporal profiles in (A), compared within the same animals (p = 0.0294, n = 7). C, Temporal profiles of IOS responses evoked by 100 ms (grey, n = 7) and 1000 ms of ChR2 stimulation (black, n = 7). D, Peak amplitudes of temporal profiles in (C), compared within the same animals (p = 0.0012, n = 7).

To determine whether ChR2-evoked hemodynamic responses were graded according to stimulus duration, as has been observed for sensation-evoked hemodynamic responses [23] and for ChR2-evoked BOLD responses [8], we examined the effect of increasing stimulus train duration on peak changes in IOS and speckle contrast (Figure 2). We found that maximal changes in speckle contrast were significantly greater (Figure 2A,B; p = 0.0294, ratio t-test of log-transformed values, n = 7) when stimulated with 1000 ms trains (5.5±1.3%, n = 7) compared to 100 ms trains (2.5±0.5%, n = 7). Correspondingly, evoked changes in IOS were greater (Figure 2C,D; p = 0.0012, paired t-test, n = 7) in response to 1000 ms stimulation (ΔI/I −0.25±0.04%, n = 7) than to 100 ms stimulation (ΔI/I −0.06±0.01%, n = 7). Maximal changes in IOS (635 nm reflectance) peaked at 1.3±0.2 s after stimulus onset (100 ms ChR2 stimulus train), while maximal changes in speckle contrast peaked at 1.1±0.3 s (paired t-test, p = 0.7188, n = 6, one animal was excluded as the peak occurred during the stimulus artifact).

**Figure 3:**
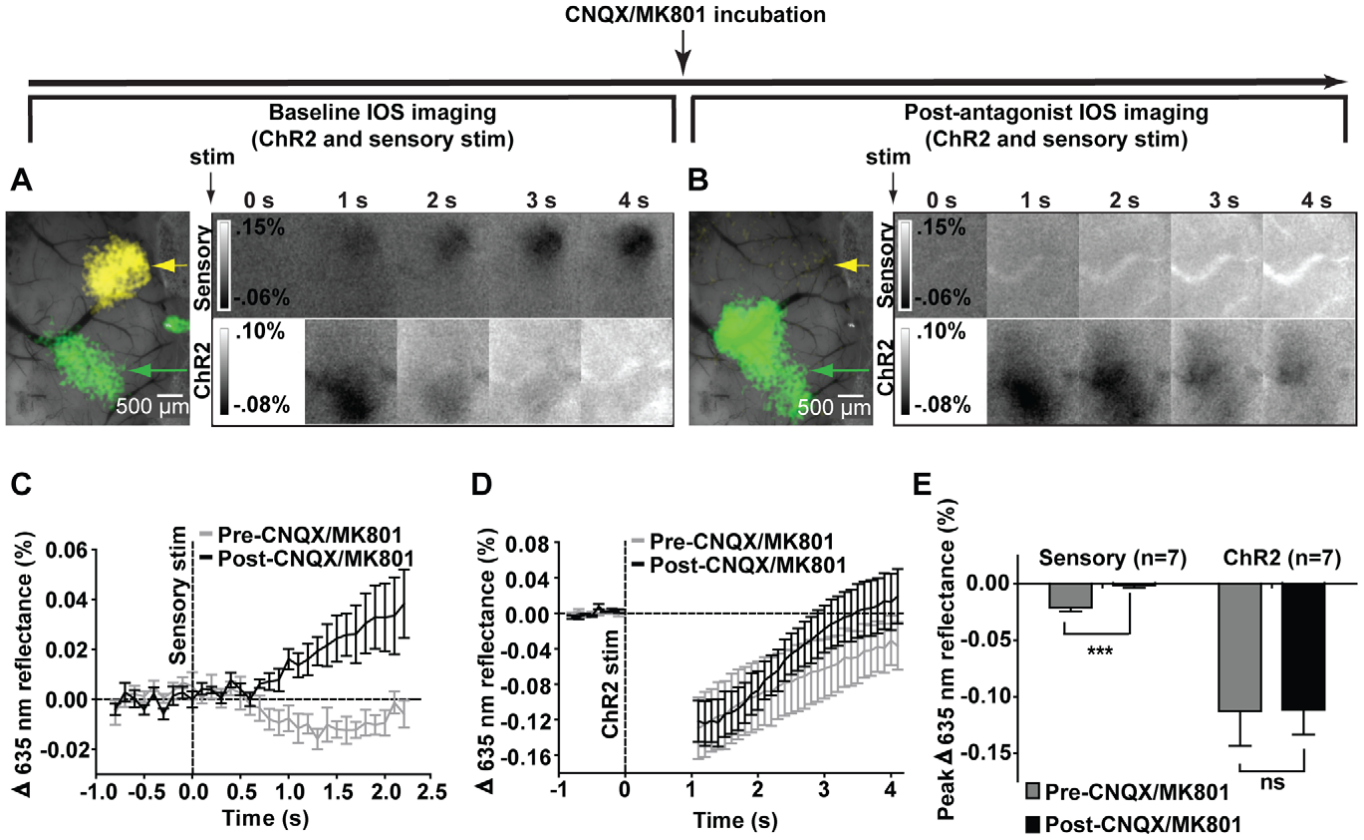
Blockade of synaptic transmission by CNQX and MK801 inhibits forepaw stimulation responses but not ChR2. A, *left*: Pre-antagonist IOS responses to sensory stimulation (yellow, indicated by yellow arrow) and ChR2 stimulation (green, indicated by green arrow) thresholded to 50% of maximum and superimposed on an image of the cortical surface. *Right*: Temporal evolution of IOS responses to 1 s of sensory stimulation (*upper*) and ChR2 stimulation (*lower*). B, *left*: Post-antagonist IOS response to sensory stimulation is inhibited (yellow arrow indicates pre-antagonist map location), while the IOS response to ChR2 stimulation is preserved (green, indicated by green arrow). *Right*: Temporal evolution of IOS responses to 1 s of sensory stimulation (*upper*) and ChR2 stimulation (*lower*). A,B Shown are the averaged results of 20 trials from a representative animal; blanked data at 0 s represents stimulus artifact. C, Temporal profiles of IOS responses to 1 s of sensory stimulation before and after CNQX/ MK801 incubation (n = 7); responses are displayed until pre-CNQX/MK801 responses return to baseline. D, Temporal profiles of IOS responses to 1 s of ChR2 stimulation before and after CNQX/MK801 incubation (n = 7); blanked data indicate laser stimulus artifact. C,D Stimulus onset denoted by vertical dotted line. E, Peak amplitudes in sensory-evoked IOS responses were significantly reduced following antagonist incubation (paired *t*-test, p = 0.0009) while peak ChR2-evoked IOS responses were not (paired *t*-test, p = 0.9358). Error bars represent SEM.

Postsynaptic activity has long been considered to be a primary determinant of neurovascular coupling [24]. To test whether ionotropic excitatory synaptic transmission were required for the production of hemodynamic responses, we first measured the effects of the AMPA receptor antagonist CNQX and the NMDA receptor antagonist MK801 on the changes in IOS evoked both by forepaw stimulation and direct activation of cortical neurons through ChR2 stimulation (Figure 3A). Following cortical incubation with CNQX/MK801, the IOS response evoked by 1 s of electrical forepaw stimulation in a representative animal was blocked, while the IOS response evoked by 1 s of ChR2 stimulation was preserved (Figure 3B). Peak changes in sensory-evoked IOS responses obtained from temporal profiles in Figure 3C were significantly reduced following incubation with CNQX/MK801 (Figure 3E; paired t-test, p = 0.0009, n = 7), while no significant reduction was observed for peak changes in ChR2-evoked IOS responses obtained from temporal profiles in Figure 3D (Figure 3E; paired t-test, p = 0.9358, n = 7).

**Figure 4:**
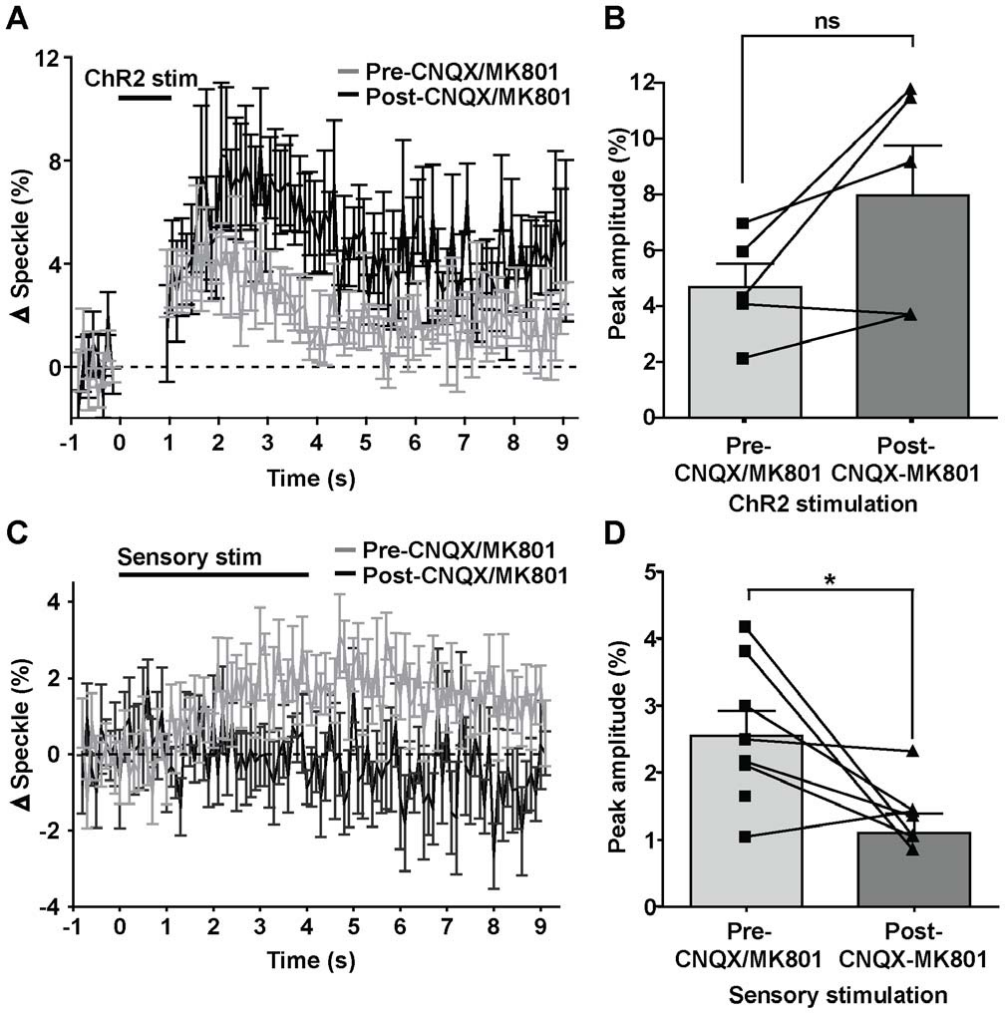
ChR2-evoked blood flow is not inhibited by blocking intracortical ionotropic glutamatergic synaptic transmission by CNQX/MK801. A, Temporal profiles of laser speckle responses evoked by 1 s of ChR2 stimulation before and after antagonist incubation. B, Comparison of peak amplitudes (averaged over 1 s) in laser speckle contrast responses in (A) before and after antagonist incubation. No significant difference was observed (n = 5, paired t-test, p = 0.0789). C, Temporal profiles of laser speckle responses evoked by forepaw sensory stimulation before and after antagonist incubation. D, Comparison of peak amplitudes (averaged over 1 s) in laser speckle contrast responses in (C) before and after antagonist incubation. A significant difference was observed (n = 8, paired t-test, p = 0.0144). Error bars represent SEM.

To investigate the effect of intracortical ionotropic excitatory synaptic transmission blockade on ChR2-evoked changes in blood flow, we examined changes in speckle contrast before and after incubation with CNQX/MK801 (Figure 4). We observed that channelrhodopsin-evoked laser speckle changes were intact following the blockade of ionotropic intracortical synaptic transmission in 5 animals (Figure 4A), with no significant difference observed in the peak speckle contrast changes (Figure 4B; paired t-test, p = 0.0789, n = 5). As expected, forepaw sensory-evoked changes in speckle contrast were significantly decreased upon incubation with CNQX/MK801 (Figure 4C; F(1,700) = 155.67; repeated measures ANOVA; p<0.0001, n = 8), with a significant reduction in peak speckle contrast (Figure 4D; paired t-test, p = 0.0144, n = 8). These results indicate that activation of neurons through ChR2 leads to increases in blood flow that can occur independently of ionotropic glutamatergic intracortical synaptic transmission.

To determine whether cortical incubation of CNQX and MK801 permitted diffusion of the antagonists to lower cortical layers, we combined antagonist incubation with laminar probe (NeuroNexus Technologies, Inc., Ann Arbor, MI) recordings at depths up to 800 µm in the forepaw sensory region. We confirmed decreases in forepaw sensory-evoked local field potential amplitude at all recorded depths. LFP deflections evoked by forepaw stimulation were not significantly larger than fluctuations recorded in the absence of stimulation before CNQX and MK801 application (RM-ANOVA, n = 7 mice; Harrison and Murphy unpublished observations).

**Figure 5:**
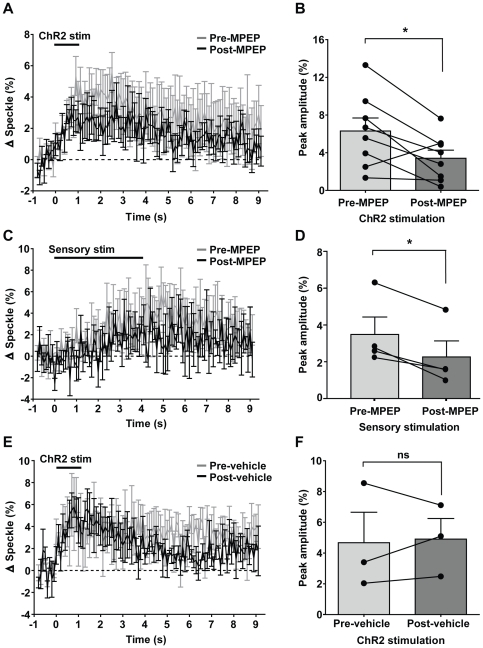
ChR2-evoked and sensory-evoked changes in blood flow are partially reduced by the mGluR5 antagonist MPEP. A, Temporal profiles of laser speckle responses evoked by 1 s of ChR2 stimulation before and after antagonist incubation. B, Comparison of peak amplitudes (averaged over 1 s) in laser speckle contrast responses in (A) before and after antagonist incubation. A significant difference was observed (n = 8, paired t-test, p = 0.0251). C, Temporal profiles of laser speckle responses evoked by sensory stimulation before and after antagonist incubation. D, Comparison of peak amplitudes (averaged over 1 s) in laser speckle contrast responses in (C) before and after antagonist incubation. A significant difference was observed (n = 4, paired t-test, p = 0.0155). E, Temporal profiles of laser speckle responses evoked by ChR2 stimulation before and after injection of saline vehicle. F, Comparison of peak amplitudes (averaged over 1 s) in laser speckle contrast responses in (E) before and after saline injection. No significant difference was observed (n = 3, paired t-test, p = 0.8211). Error bars represent SEM.

In vitro experiments have suggested that the astrocytic Group I metabotropic glutamate receptor mGluR5 couples presynaptic release of glutamate to changes in blood flow [25], however other in vivo results in rodent somatosensory cortex suggest no significant effect [26]. Our results with ChR2 stimulation indicate only a partial reduction in ChR2-evoked speckle contrast following a 20 min incubation of the mGluR5 antagonist MPEP (Figure 5A; repeated measures ANOVA, F(1,700) = 59.62, p<0.0001, n = 8). The peak ChR2-evoked speckle contrast was significantly reduced (Figure 5B; paired t-test, p = 0.0251, n = 8) from a pre-MPEP mean of 6.3±1.4% to a post-MPEP mean of 3.4±0.9%, resulting in a mean reduction of 33.7±20.8% when compared over all 8 animals. Half of the animals used for ChR2 stimulation were also examined for the effect of MPEP incubation on sensory-evoked speckle contrast, measured at 40 min post-MPEP incubation. MPEP incubation resulted in a significant reduction in forepaw sensory-evoked speckle contrast (Figure 5C; F(1,300) = 81.82, p<0001; two-way repeated measures ANOVA, n = 4), with a significant (Figure 5D; p = 0.0155, t-test, n = 4) reduction in peak speckle contrast observed, from a pre-drug mean of 3.5±0.9% to a post-drug mean of 2.3±0.9%, resulting in a mean reduction of 41.97±7.40% overall. To test whether this reduction were attributable to the injection itself, we compared ChR2-evoked speckle contrast before and after injection of the saline vehicle alone. No significant difference was observed (Figure 5 E,F; paired t-test, p = 0.8211, n = 3) from a pre-vehicle mean of 4.7±2.0% to a post-vehicle mean of 4.9±1.3%.

To determine whether there were non-specific effects of MPEP, we examined its effect on unstimulated baseline EEG (Figure S3A,B). We examined 10 min of spontaneous EEG collected before and after MPEP injection (up to 20 min post-injection). In 4 animals, power spectra amplitudes were compared by frequency band (Figure S3B) before and after MPEP incubation, and no significant effect of MPEP was observed (Bonferroni post-test, all p>0.05, n = 4). In order to determine whether the observed reductions in blood flow responses upon MPEP incubation could be attributable to changes in underlying physiology, we compared heart rate and oxygen saturation recordings prior to MPEP incubation and at 0 min and 10 min post-MPEP incubation. No significant effect of drug incubation was observed (Figure S3C; repeated measures ANOVA, p = 0.2308, F(2,8) = 1.77, n = 3), nor when all post-drug time points were compared to pre-drug averages (all p<0.05 for oxygen saturation and heart rate, n = 3).

## Discussion

We have demonstrated that stimulation of ChR2-expressing pyramidal neurons is sufficient to evoke changes in oxygenation and blood flow and further, that production of these hemodynamic signals is not sensitive to reduction of intracortical excitatory synaptic transmission through ionotropic glutamate receptors.

### Ionotropic glutamate receptor-independent mechanisms of neurovascular coupling

The predominant view is that brain activity dependent changes in oxygenation [27], [28] and cerebral blood flow [29], [30] are strongly coupled to local glutamate-dependent postsynaptic neuronal activity, both in the cortex and cerebellum. To date, the only exceptions to this have been observed in the olfactory bulb, where changes in IOS [20] and blood flow [31] were evoked in the absence of ionotropic glutamate receptor activation. However, as argued by Attwell et al. (2010) [1], much of the pharmacological and electrophysiological support ascribed towards ionotropic glutamate receptor dependency applies equally to glutamate-activated astrocytic pathways. Indeed, one of the key pieces of evidence [32] in favour of postsynaptic neuronal signaling – the correlation of local field potentials (reflecting input and intracortical processing) with changes in blood flow – could be similarly correlated with astrocyte activation. However, within the in vivo situation it would be difficult to extricate glial cells from the circuits of the brain [2].

The fact that ChR2-evoked changes in blood flow showed a trend towards increased magnitude upon blockade of ionotropic glutamate receptors is suggestive of interneuron-mediated inhibition through tonic activation of presynaptic GABA-B receptors, as observed in the olfactory bulb [31], [33]; thus, the increased blood flow we observed could point to enhanced activation of metabotropic glutamate receptors through elevated presynaptic release of glutamate. Our results suggest that ChR2-evoked neuronal activity, as with sensory-evoked activity, is coupled partially (∼34%) to functional hyperemia via an mGluR5-dependent pathway [25]. Similarly, studies of the olfactory bulb [31] and cortex [34] have demonstrated a partial role for presynaptic release of glutamate in triggering hemodynamic responses via metabotropic glutamate receptors, in particular mGluR5, while other data suggests no significant role of mGluR5 in rodent somatosensory cortex [26]. Why is the mGluR5 effect on ChR2-evoked elevation in blood flow only partial? As observed by [35] in vitro, only a relatively small fraction of astrocytes consistently responded to ChR2 activation within neurons, a number that was subsequently reduced upon mGluR5 blockade by MPEP. Thus, it is possible that the partial reduction we observe represents the full blockade of mGluR5 receptors, but that the remainder of the response operates via alternate pathways, possibly involving astrocytic glutamate uptake transporters [20].

In summary, we have demonstrated the ability to stimulate changes in oxygenation and blood flow through direct activation of excitatory cortical neurons. Moreover, we have provided evidence that neuronal or glial ionotropic glutamate receptors may not need to be the primary mediators of neurovascular coupling initiated by direct neuronal activation. However, we acknowledge that our findings do not preclude alternate mechanisms involving synaptic activation of intermediary neuronal populations or glia that do not involve CNQX- or MK-801-sensitive glutamate receptors. Alternatively, it is possible that vasoactive agents [36], released as a consequence of ChR2-initiated action potentials may act upon nearby capillaries or arterioles to produce changes in dilation and concomitant changes in blood flow, thus coupling activity in activated neurons to the surrounding vasculature.

## Supporting Information

Figure S1 **No channelrhodopsin-evoked hemodynamic responses are observed in non-channelrhodopsin-expressing and dead channelrhodopsin-positive animals.** A, (left) Green light image of the cortical surface, peak IOS response (averaged over 1 s from peak) (middle) and peak normalized laser speckle contrast response (averaged over 1 s at peak) (right) to 1 s of ChR2 stimulation; data shown are averaged 20 trials from a representative animal. Temporal profiles of (B) IOS and (C) laser speckle responses evoked by ChR2 stimulation for ChR2-negative and ChR2-positive animals. Temporal profiles of (D) IOS and (E) laser speckle responses evoked by ChR2 stimulation for dead ChR2-positive mice. B–E, Black bars represent onset and duration of stimulation. Blanked data in temporal profiles reflect laser stimulus artifact. Error bars represent SEM, all n = 2.
(PDF)

Figure S2 **Effect of ChR2 stimulation on spontaneous EEG activity, sensory-evoked IOS responses and baseline physiology.** A, Temporal profiles of sensory-evoked IOS responses evoked before (grey) and after (black) 20 trials of ChR2 stimulation, as shown in timeline (above). B, Peak responses of temporal profiles in (A) before and after ChR2 stimulation. No significant difference was observed (n = 5, p = 0.3463, paired t-test). C, Power spectrum analysis of spontaneous EEG recordings before (grey) and after (black) 20 trials of ChR2 stimulation. D, Power spectra in (C) compared by frequency band; bands Alpha-Gamma are graphed on the right y-axis. C,D n = 6, all p>0.05. E, ChR2 stimulation had no significant effect on either mean heart rate and oxygen saturation measurements (Bonferroni post-test, both p>0.05) during 20 trials of ChR2 stimulation in comparison to measurements obtained prior to ChR2 stimulation (repeated measures ANOVA, F(1,6) = 0.01, p = 0.9384), although ChR2 did have a significant effect on the variance of oxygen saturation (p = 0.0365).
(PDF)

Figure S3 **Effect of MPEP on spontaneous EEG activity and baseline physiology.** A, Power spectrum analysis of spontaneous EEG recordings before (grey) and after (black) MPEP injection. B, Power spectra in (A) compared by frequency band; bands Alpha-Gamma are graphed on the right y-axis. No significant effect of MPEP incubation was observed for any frequency band (Bonferroni post-test, all p>0.05, n = 4). C, Heart rate and oxygen saturation recordings prior to MPEP incubation and at 0 minutes and 10 minutes post-MPEP incubation. No significant effect of drug incubation was observed (repeated measures ANOVA, p = 0.2308, F(2,8) = 1.77, n = 3), nor when all post-drug time points were compared to pre-drug averages (all p<0.05 for oxygen saturation and heart rate, n = 3).
(PDF)

## References

[1] Attwell D, Buchan AM, Charpak S, Lauritzen M, Macvicar BA (2010). Glial and neuronal control of brain blood flow.. Nature.

[2] Petzold GC, Murthy VN (2011). Role of astrocytes in neurovascular coupling.. Neuron.

[3] Wang H, Peca J, Matsuzaki M, Matsuzaki K, Noguchi J (2007). High-speed mapping of synaptic connectivity using photostimulation in Channelrhodopsin-2 transgenic mice.. Proc Natl Acad Sci U S A.

[4] Arenkiel BR, Peca J, Davison IG, Feliciano C, Deisseroth K (2007). In vivo light-induced activation of neural circuitry in transgenic mice expressing channelrhodopsin-2.. Neuron.

[5] Ayling OG, Harrison TC, Boyd JD, Goroshkov A, Murphy TH (2009). Automated light-based mapping of motor cortex by photoactivation of channelrhodopsin-2 transgenic mice.. Nat Methods.

[6] Feng GP, Mellor RH, Bernstein M, Keller-Peck C, Nguyen QT (2000). Imaging neuronal subsets in transgenic mice expressing multiple spectral variants of GFP.. Neuron.

[7] Desai M, Kahn I, Knoblich U, Bernstein J, Atallah H (2011). Mapping brain networks in awake mice using combined optical neural control and fMRI.. J Neurophysiol.

[8] Lee JH, Durand R, Gradinaru V, Zhang F, Goshen I (2010). Global and local fMRI signals driven by neurons defined optogenetically by type and wiring.. Nature.

[9] Tolias AS, Sultan F, Augath M, Oeltermann A, Tehovnik EJ (2005). Mapping cortical activity elicited with electrical microstimulation using FMRI in the macaque.. Neuron.

[10] Dunn JF, Tuor UI, Kmech J, Young NA, Henderson AK (2009). Functional brain mapping at 9.4T using a new MRI-compatible electrode chronically implanted in rats.. Magn Reson Med.

[11] Zhang S, Murphy TH (2007). Imaging the impact of cortical microcirculation on synaptic structure and sensory-evoked hemodynamic responses in vivo.. PLoS Biol.

[12] Devor A, Hillman EM, Tian P, Waeber C, Teng IC (2008). Stimulus-induced changes in blood flow and 2-deoxyglucose uptake dissociate in ipsilateral somatosensory cortex.. J Neurosci.

[13] Vanzetta I, Grinvald A (2008). Coupling between neuronal activity and microcirculation: implications for functional brain imaging.. HFSP J.

[14] Frostig RD, Lieke EE, Ts'o DY, Grinvald A (1990). Cortical functional architecture and local coupling between neuronal activity and the microcirculation revealed by in vivo high-resolution optical imaging of intrinsic signals.. Proc Natl Acad Sci U S A.

[15] Harrison TC, Sigler A, Murphy TH (2009). Simple and cost-effective hardware and software for functional brain mapping using intrinsic optical signal imaging.. J Neurosci Methods.

[16] Briers JD (2001). Laser Doppler, speckle and related techniques for blood perfusion mapping and imaging.. Physiol Meas.

[17] Dunn AK, Bolay H, Moskowitz MA, Boas DA (2001). Dynamic imaging of cerebral blood flow using laser speckle.. J Cereb Blood Flow Metab.

[18] Sigler A, Goroshkov A, Murphy TH (2008). Hardware and methodology for targeting single brain arterioles for photothrombotic stroke on an upright microscope.. J Neurosci Methods.

[19] Cheng H, Duong TQ (2007). Simplified laser-speckle-imaging analysis method and its application to retinal blood flow imaging.. Opt Lett.

[20] Gurden H, Uchida N, Mainen ZF (2006). Sensory-evoked intrinsic optical signals in the olfactory bulb are coupled to glutamate release and uptake.. Neuron.

[21] Grinvald A, Lieke E, Frostig RD, Gilbert CD, Wiesel TN (1986). Functional architecture of cortex revealed by optical imaging of intrinsic signals.. Nature.

[22] Hira R, Honkura N, Noguchi J, Maruyama Y, Augustine GJ (2009). Transcranial optogenetic stimulation for functional mapping of the motor cortex.. J Neurosci Methods.

[23] Nemoto M, Sheth S, Guiou M, Pouratian N, Chen JW (2004). Functional signal- and paradigm-dependent linear relationships between synaptic activity and hemodynamic responses in rat somatosensory cortex.. J Neurosci.

[24] Attwell D, Laughlin SB (2001). An energy budget for signaling in the grey matter of the brain.. J Cereb Blood Flow Metab.

[25] Zonta M, Angulo MC, Gobbo S, Rosengarten B, Hossmann KA (2003). Neuron-to-astrocyte signaling is central to the dynamic control of brain microcirculation.. Nat Neurosci.

[26] Calcinaghi N, Jolivet R, Wyss MT, Ametamey SM, Gasparini F (2011). Metabotropic glutamate receptor mGluR5 is not involved in the early hemodynamic response.. J Cereb Blood Flow Metab.

[27] Caesar K, Hashemi P, Douhou A, Bonvento G, Boutelle MG (2008). Glutamate receptor-dependent increments in lactate, glucose and oxygen metabolism evoked in rat cerebellum in vivo.. J Physiol.

[28] Lecoq J, Tiret P, Najac M, Shepherd GM, Greer CA (2009). Odor-evoked oxygen consumption by action potential and synaptic transmission in the olfactory bulb.. J Neurosci.

[29] Norup Nielsen A, Lauritzen M (2001). Coupling and uncoupling of activity-dependent increases of neuronal activity and blood flow in rat somatosensory cortex.. J Physiol.

[30] Chaigneau E, Tiret P, Lecoq J, Ducros M, Knopfel T (2007). The relationship between blood flow and neuronal activity in the rodent olfactory bulb.. J Neurosci.

[31] Petzold GC, Albeanu DF, Sato TF, Murthy VN (2008). Coupling of neural activity to blood flow in olfactory glomeruli is mediated by astrocytic pathways.. Neuron.

[32] Logothetis NK, Pauls J, Augath M, Trinath T, Oeltermann A (2001). Neurophysiological investigation of the basis of the fMRI signal.. Nature.

[33] Pirez N, Wachowiak M (2008). In vivo modulation of sensory input to the olfactory bulb by tonic and activity-dependent presynaptic inhibition of receptor neurons.. J Neurosci.

[34] Takano T, Tian GF, Peng W, Lou N, Libionka W (2006). Astrocyte-mediated control of cerebral blood flow.. Nat Neurosci.

[35] Bernardinelli Y, Salmon C, Jones EV, Farmer WT, Stellwagen D (2011). Astrocytes display complex and localized calcium responses to single-neuron stimulation in the hippocampus.. J Neurosci.

[36] Hamel E (2006). Perivascular nerves and the regulation of cerebrovascular tone.. J Appl Physiol.

