# Supplementary figures and images for: Hemodynamic Responses Evoked by Neuronal Stimulation via Channelrhodopsin-2 Can Be Independent of Intracortical Glutamatergic Synaptic Transmission
Source: PLoS One. 2012 Jan 10;7(1):e29859. doi: 10.1371/journal.pone.0029859 (PMC3254633; doi:10.1371/journal.pone.0029859)

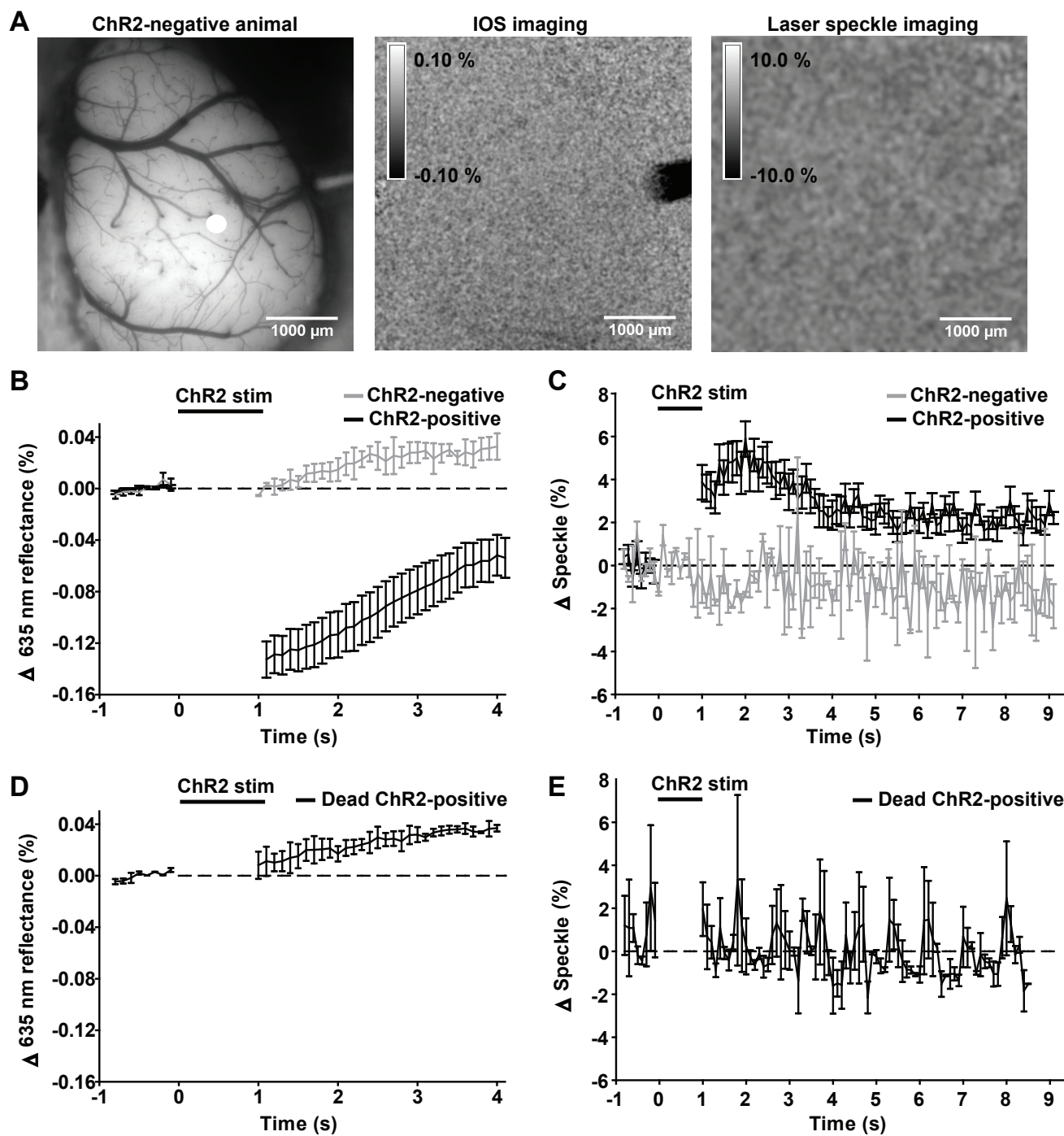

Figure S1

Supplement: Figure S1 — No channelrhodopsin-evoked hemodynamic responses are observed in non-channelrhodopsin-expressing and dead channelrhodopsin-positive animals. A, (left) Green light image of the cortical surface, peak IOS response (averaged over 1 s from peak) (middle) and peak normalized laser speckle contrast response (averaged over 1 s at peak) (right) to 1 s of ChR2 stimulation; data shown are averaged 20 trials from a representative animal. Temporal profiles of (B) IOS and (C) laser speckle responses evoked by ChR2 stimulation for ChR2-negative and ChR2-positive animals. Temporal profiles of (D) IOS and (E) laser speckle responses evoked by ChR2 stimulation for dead ChR2-positive mice. B–E, Black bars represent onset and duration of stimulation. Blanked data in temporal profiles reflect laser stimulus artifact. Error bars represent SEM, all n = 2. (PDF) [file pone.0029859.s001.pdf]

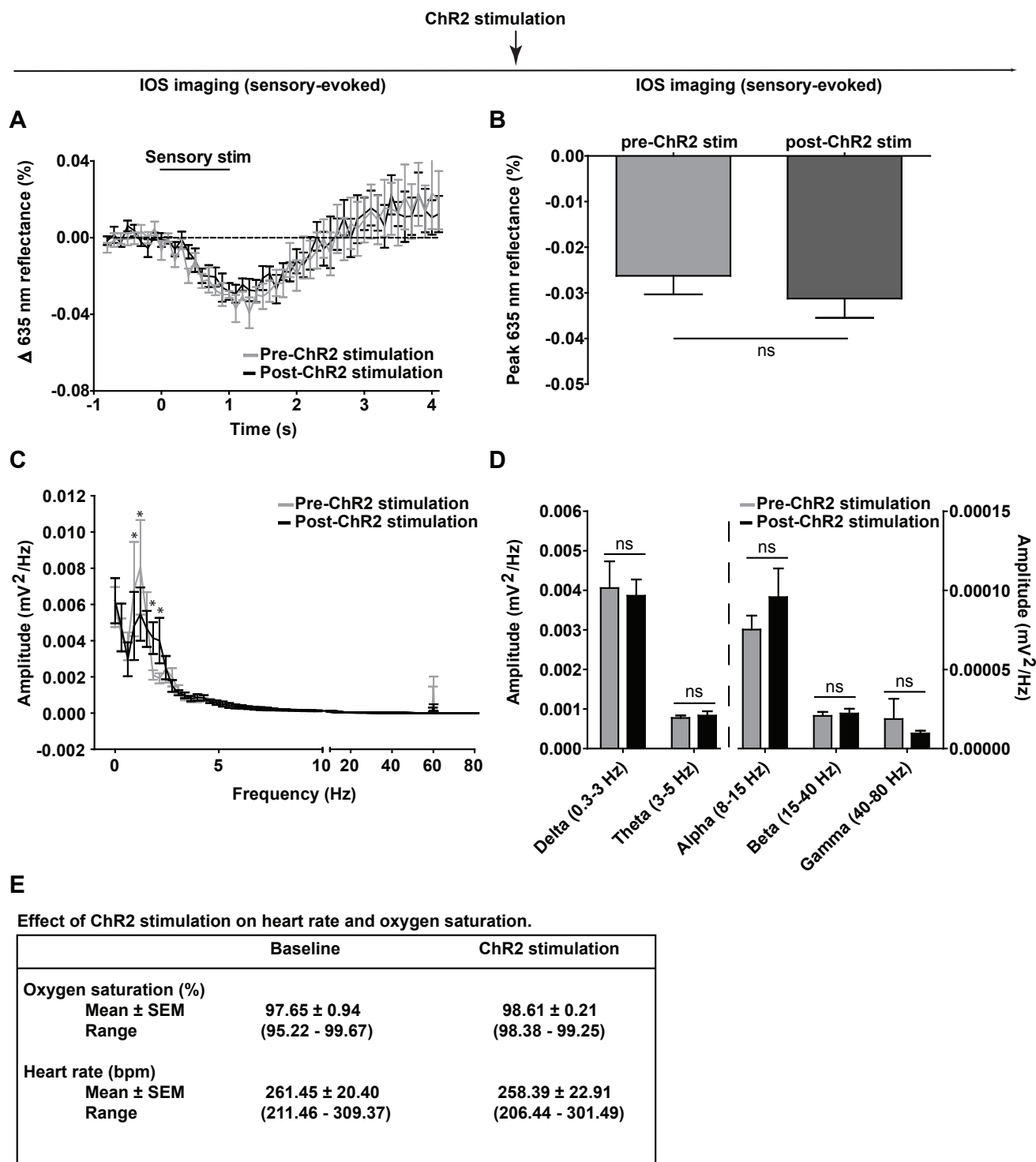

Figure S2

Supplement: Figure S2 — Effect of ChR2 stimulation on spontaneous EEG activity, sensory-evoked IOS responses and baseline physiology. A, Temporal profiles of sensory-evoked IOS responses evoked before (grey) and after (black) 20 trials of ChR2 stimulation, as shown in timeline (above). B, Peak responses of temporal profiles in (A) before and after ChR2 stimulation. No significant difference was observed (n = 5, p = 0.3463, paired t-test). C, Power spectrum analysis of spontaneous EEG recordings before (grey) and after (black) 20 trials of ChR2 stimulation. D, Power spectra in (C) compared by frequency band; bands Alpha-Gamma are graphed on the right y-axis. C,D n = 6, all p>0.05. E, ChR2 stimulation had no significant effect on either mean heart rate and oxygen saturation measurements (Bonferroni post-test, both p>0.05) during 20 trials of ChR2 stimulation in comparison to measurements obtained prior to ChR2 stimulation (repeated measures ANOVA, F(1,6) = 0.01, p = 0.9384), although ChR2 did have a significant effect on the variance of oxygen saturation (p = 0.0365). (PDF) [file pone.0029859.s002.pdf]

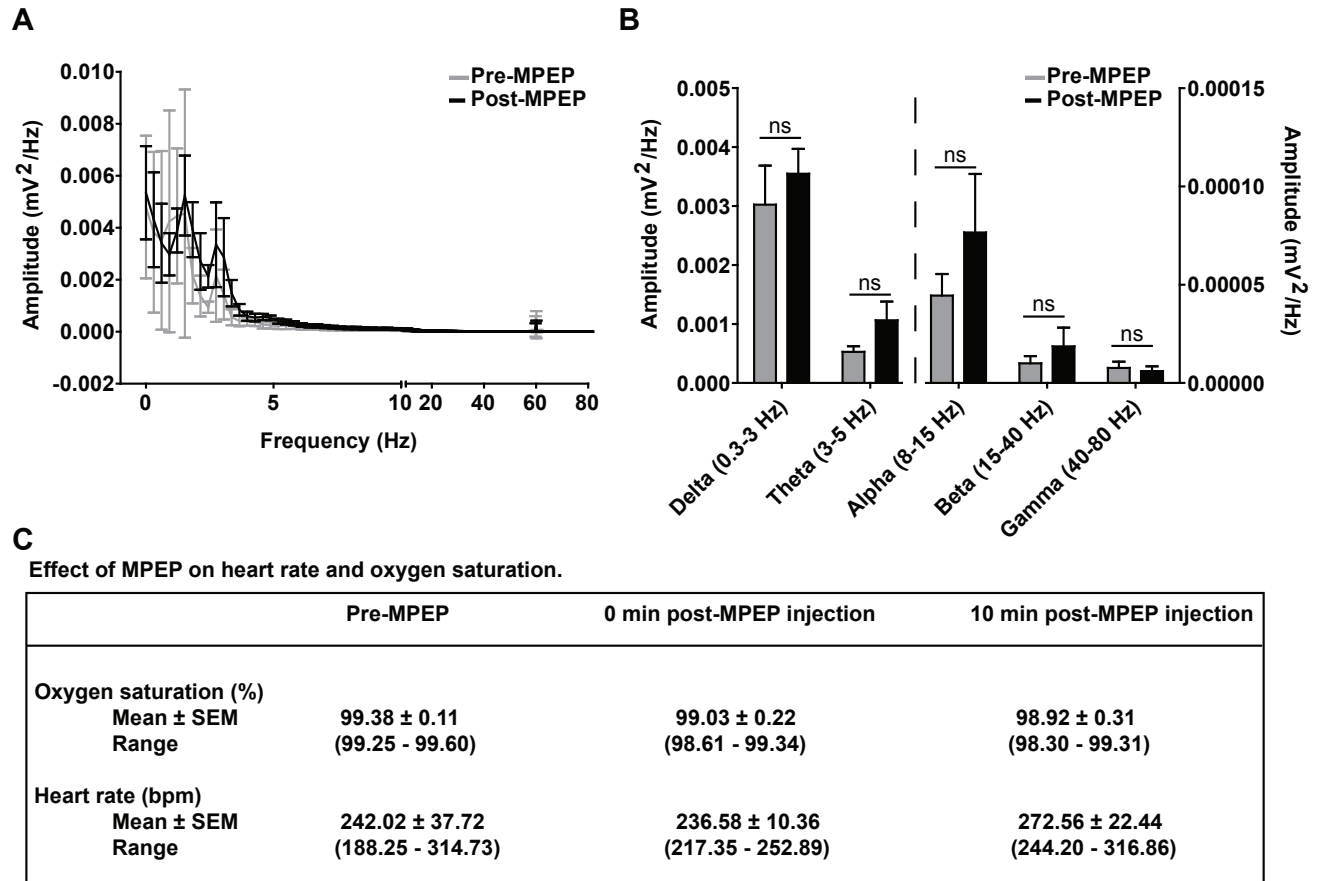

Figure S3

Supplement: Figure S3 — Effect of MPEP on spontaneous EEG activity and baseline physiology. A, Power spectrum analysis of spontaneous EEG recordings before (grey) and after (black) MPEP injection. B, Power spectra in (A) compared by frequency band; bands Alpha-Gamma are graphed on the right y-axis. No significant effect of MPEP incubation was observed for any frequency band (Bonferroni post-test, all p>0.05, n = 4). C, Heart rate and oxygen saturation recordings prior to MPEP incubation and at 0 minutes and 10 minutes post-MPEP incubation. No significant effect of drug incubation was observed (repeated measures ANOVA, p = 0.2308, F(2,8) = 1.77, n = 3), nor when all post-drug time points were compared to pre-drug averages (all p<0.05 for oxygen saturation and heart rate, n = 3). (PDF) [file pone.0029859.s003.pdf]
